# A Mobile Smoking Cessation Intervention for Mexico (Vive sin Tabaco... ¡Decídete!): Single-Arm Pilot Study

**DOI:** 10.2196/12482

**Published:** 2019-04-25

**Authors:** Ana Paula Cupertino, Francisco Cartujano-Barrera, Mariana Ramírez, Rosibel Rodríguez-Bolaños, James F Thrasher, Gloria Pérez-Rubio, Ramcés Falfán-Valencia, Edward F Ellerbeck, Luz Myriam Reynales-Shigematsu

**Affiliations:** 1 Department of Cancer Prevention and Control Hackensack University Medical Center Hackensack, NJ United States; 2 Department of Preventive Medicine and Public Health University of Kansas Medical Center Kansas City, KS United States; 3 Department of Tobacco Research National Institute of Public Health Cuernavaca Mexico; 4 Department of Health Promotion, Education and Behavior University of South Carolina Columbia, SC United States; 5 Instituto Nacional de Enfermedades Respiratorias Ismael Cosio Villegas Laboratorio HLA Mexico City Mexico

**Keywords:** smoking, smoking cessation, mHealth, text messages, global health

## Abstract

**Background:**

Of the 14.3 million Mexicans who smoke, only a minority take advantage of evidence-based approaches to smoking cessation. Mobile health interventions have the potential to increase the reach of effective cessation interventions in Mexico.

**Objective:**

This study aimed to assess the feasibility and acceptability of an innovative, personalized, and interactive smoking cessation mobile intervention developed for Mexican smokers.

**Methods:**

We recruited 40 Mexican smokers to participate in *Vive sin Tabaco... ¡Decídete!*, a smoking cessation program that uses a tablet-based decision support software to drive a 12-week text messaging smoking cessation program and pharmacotherapy support. Outcome measures included participant text messaging interactivity with the program, participant satisfaction, and 12-week verified abstinence using urinary cotinine testing or exhaled carbon monoxide.

**Results:**

Average age of the participants was 36 years (SD 10.7), and they were primarily male (65%, 26/40) with at least an undergraduate degree (62%, 25/40). Most participants (95%, 38/40) smoked daily and were interested in quitting in the next 7 days. As an indicator of participant interactivity, participants sent an average of 21 text messages during the 12-week intervention (SD 17.62). Of the 843 messages that participants sent to the program, only 96 messages (11.3%, 96/843) used keywords. At 12 weeks, 40% (16/40) of participants were biochemically verified (87%, 35/40, follow-up rate). The majority of participants (85%, 30/35) reported being very satisfied or extremely satisfied with the program.

**Conclusions:**

The *Vive sin Tabaco... ¡Decídete!* smoking cessation mobile intervention was accepted by participants, generated high satisfaction and high text messaging interactivity, and resulted in a noteworthy cessation rate at the end of treatment. This intervention is a promising strategy for smoking cessation in Mexico. Additional testing as a formal randomized clinical trial appears warranted.

## Introduction

### Background

Currently, 14.3 million Mexican adults (16.4%) smoke [1], and it is expected that more than 4 million will die of tobacco-related diseases in the next decade if smoking prevalence remains unchecked [2]. The prevalence of smoking has remained relatively consistent over the last decade [1,3], despite implementation of taxes [4,5], smoke-free policies [6,7], health warnings on cigarette packages [8-10], and advertising restrictions [11], as recommended by the World Health Organization’s Framework Convention on Tobacco Control [12]. The potential for reducing the projected morbidity and mortality associated with smoking depends greatly on reaching smokers and delivering cost-effective cessation interventions.

Currently, 8 in 10 Mexican smokers are interested in quitting smoking [1]. However, less than 10% of Mexican smokers take advantage of the evidence-based approaches to smoking cessation (pharmacotherapy and counseling) [1] that are offered by the public health care system [13-15]. Although health care providers in Mexico are encouraged to address smoking with their patients in every visit [16,17], most health care providers fail to initiate cessation treatment [18]. Overcoming the burden of tobacco use in Mexico demands affordable, accessible, and effective solutions.

Mexicans are more likely to be nondaily and light smokers (<10 cigarettes per day [CPD]) [1,19]. The 2015 Global Adult Tobacco Survey in Mexico found that 53.7% of current smokers are nondaily smokers and, among daily smokers, the average number of CPD was 7.7 [1]. Light smokers believe their lower level of smoking reduces or eliminates their health risk despite evidence to the contrary [20]. Light smoking significantly increases the risk for cancer, all-cause mortality, and adverse cardiovascular outcomes [21]. It is important to identify innovative smoking cessation strategies tailored to the needs of Mexican smokers.

Developments in the sophistication of mobile technologies allow for flexible delivery of text messages, with algorithms used to tailor content to individual motivational and behavioral needs for smoking cessation [22-26]. A number of studies have examined the effectiveness of text messaging interventions to promote and support smoking cessation [27-29]. A Cochrane meta-analysis of these studies indicates that text messaging interventions increase the likelihood of staying quit by approximately 1.7 times (9.3% quit rate with text messages vs 5.6% quit rate with no program) [27]. All the studies included in the meta-analysis were conducted in high-income countries with limited generalizability to low- and middle-income countries. Reflecting the global trend in the uptake of cell phones, Mexico is the eleventh largest mobile market in the world, with 107.8 million active cell phones [30] for its 123 million inhabitants [31]. The very high rate of cell phone ownership, the low use of smoking cessation treatments, and the evidence that text messaging can enhance a smoking cessation intervention provide a unique opportunity to assess a smoking cessation mobile intervention in Mexico. Considering this premise, the US National Cancer Institutes’ text messaging program [32] was adapted for Mexican smokers interested in quitting using focus groups and interviews [33]; however, no formal evaluation of that effort has been done.

### Objective

This pilot study aimed to assess the feasibility and acceptability of *Vive sin Tabaco... ¡Decídete!*, an innovative smoking cessation mobile intervention for Mexican smokers. This mobile innovation is achieved by connecting a Web-based decision-making tool used to develop a personalized quit plan with the delivery of tailored text messages over 12 weeks. Results from this study will inform future implementation and dissemination studies to achieve significant reductions in tobacco-related morbidity and mortality in Mexico and provide a model for population-based smoking cessation mobile interventions.

## Methods

### Setting

This study was conducted between March and August 2017 at the Medical Center of the Autonomous University of the State of Morelos, located in Cuernavaca, Morelos, Mexico. This urban primary health care clinic serves an average of 100 individuals on a daily basis. None of the services provided by the clinic address smoking cessation.

### Participants

Participants were recruited through printed posters and multimedia venues including ads through the National Institute of Public Health’s website and Facebook and local radio announcements. Potential participants emailed or called the study personnel to learn more about the study. Eligibility assessment was conducted over the phone. Eligible participants were of Mexican origin, aged 18 years or older, had smoked for at least 6 months, smoked at least 3 days per week, were interested in quitting within the next 30 days, had a cell phone with text messaging capacity, and were willing to complete baseline and 12-week follow-up surveys. Participants were excluded from the study if they were planning to move within the next 6 months, consumed other forms of tobacco (including electronic cigarettes) or illicit drugs in Mexico (eg, cannabis and cocaine), or had another household member enrolled in the study. All subjects gave informed consent before participation in the study. Participants received 300 Mexican pesos (approximately US $17) at baseline and follow-up as an incentive for their time and transportation. The Human Subjects Committee of the National Institute of Public Health approved the study procedures.

### Intervention

*Vive sin Tabaco... ¡Decídete!* is a smoking cessation mobile intervention that encompasses 3 integrated components: (1) a tablet-based software that collects personal smoking-related information to support the development of an individualized quit plan and guides the ensuing text messages program, (2) a 12-week individually tailored text messages program with interactive capabilities, and (3) pharmacotherapy support when applicable.

#### Vive sin Tabaco... ¡Decídete! Tablet-Based Software

The tablet-based, decision support tool was designed to help smokers create a personalized smoking cessation plan and to collect data that tailored the text messages delivered over the ensuing 12 weeks [34]. This tool was adapted from 2 smoking cessation Web-based, informed decision-making tools for Latinos in the United States [35] and Mexico [36,37]. This tablet-based tool consisted of interactive features that lead smokers through various steps in the quitting process. The program included testimonies from ex-smokers and features short video clips (0:21-2:03 min) and narrated graphics on the benefits of quitting while also describing how cessation pharmacotherapy (nicotine replacement therapy [NRT]) can support abstinence. The program also collected basic information about the participants’ smoking history, including the number of days they smoke each week and the number of cigarettes smoked per day. At the end of the 10- to 15-min session, participants were prompted to request pharmacotherapy if interested and clinically recommended and to select a quit date within a 30-day timeframe. Upon completion of the tablet-based software, participants were provided with a 1-page summary of their personalized cessation plan (eg, the selected quit date and pharmacotherapy with the recommended dose and regimen). Next, participants automatically began receiving the text message portion of the intervention.

#### Vive sin Tabaco... ¡Decídete! Text Messaging

We developed a library of 304 text messages in Spanish to support a 12-week cessation program [38] based on the social cognitive theory [39]. Text messages were informed by literature reviews on educational facts and strategies for smoking cessation, feedback from national tobacco control experts, and results from focus groups with Mexican smokers and ex-smokers. The text message intervention allowed 3 levels of interactivity: (1) prescheduled standard messages, (2) keyword-triggered standard messages, and (3) counselor-personalized responses.

##### Prescheduled Standard Messages

The main goal of the prescheduled standard messages was to provide counseling through *educational* (eg, health risks of smoking, immediate and long-term benefits of quitting smoking, and how to correctly use the pharmacotherapy), *motivational* (eg, intrinsic and extrinsic motivation), and *behavioral* (eg, reminders to use strategies to cope with triggers, self-control through goals, self-monitoring, and prompts to order NRT) messages to facilitate quitting and supporting abstinence. Messages were automatically tailored to the participant’s name(s), gender, pharmacotherapy indication, and the selected quit date. Text messages were organized along a 12-week timeline designed to support a personalized quit plan: (1) prequit (29 days), (2) quit day (1 day), (3) maintenance (28 days), and (4) relapse prevention (8 weeks). The content and number of messages varied as the intervention progressed (see Table 1).

##### Keyword-Triggered Standard Messages

These messages consisted of automated immediate responses sent to participants who texted 1 of the following keywords: *Antojo* (Spanish for “Crave”), *Estrés* (Spanish for “Stress”), *Recaída* (Spanish for “Relapse”), *Familia* (Spanish for “Family”), *Tristeza* (Spanish for “Sadness”), and *Consejo* (Spanish for “Advice”). In addition, throughout the 12-week program, participants received 7 response-triggered (YES or NO) text messages to assess their smoking status (eg, *¿Sigues sin fumar? Responde SÍ o NO y te ayudaremos. ¡Seguimos contigo!* [Spanish for “Are you still smoke-free? Reply YES or NO and we will help you. We are here with you!”]). If participants indicated that they were smoking, these automated messages encouraged them to set a new quit date. Following the Mexican Federal Telecommunications Institute regulations, participants could withdraw from the text message program at any moment by sending the keyword *Alto* (Spanish for “Stop”) [40].

##### Counselor-Personalized Responses

Taking advantage of the text message platform’s capability to recognize free texting (nonkeyword) from participants, *Vive sin Tabaco... ¡Decídete!* encouraged participants to text any feelings, concerns, and/or questions to the program (eg, *Puedes escribirnos en todo momento. Te apreciamos y nuestro compromiso es ayudarte. ¡Recuérdalo!* [Spanish for “You can text us at any time. We appreciate you and we are committed to help you. Remember it!”]). A trained smoking cessation counselor answered these messages following standardized protocols (eg, motivational interview and pharmacotherapy delivery, use, adherence, and side effects). The counselor was trained on the Basic Skills for Working with Smokers course by The University of Massachusetts Medical School [41]. The counselor monitored and triaged queries daily, responding within 24 hours of receipt of text messages sent by participants.

**Table 1:** Types of messages, stages, duration, number, and examples of text messages.

Type of message	Stage	Duration	Number	Examples
Prescheduled standard messages	Prequit	29 days	2 to 3 messages a day	*[Nombre], crea un grupo que te apoye en este proceso, pueden ser tus familiares y amistades más cercanas. Diles que a partir de [Fecha para dejar de fumar] dejarás de fumar. ¡Cuenta con ellos(as), cuenta con nosotros!*; [Name], create a group that will support you through this process, it can be your family and close friends. Tell them that from [Quit date] you will quit smoking. Count on them, count on us!; *¡BUENAS NOTICIAS! Dejando de fumar cuidas el medio ambiente. ¿Cómo? Cada año, la industria tabacalera es responsable de la tala de 600 millones de árboles;* GOOD NEWS! By quitting smoking, you are taking care of the environment. How? Each year, the tobacco industry is responsible for cutting down 600 million trees.; *[Nombre], al dejar el cigarro te convertirás en un ejemplo para otros, ¡muchos querrán lograrlo como tú!*; [Name], when you quit smoking, you become a role model to others, many will want to stop smoking like you!; *La vida está llena de retos, dejar de fumar es uno más que vas a superar. Piensa en lo bien que te sentirás cuando lo hayas logrado*; Life is full of challenges, quitting smoking is one more challenge you will overcome. Think about how good you will feel once you achieve it!
Prescheduled standard messages	Quit day	1 day	4 messages	*[Nombre], ¡FELICIDADES! Ha llegado el día. Hoy comienzas una nueva vida. Recuerda por qué estás dejando de fumar*; [Name], CONGRATULATIONS! The day has arrived. Today you start a new life. Remember why you are quitting smoking; *[Nombre], ¿cómo te sientes? Sabemos que las primeras 24 horas son las más difíciles, ¡recuerda que cuentas con nosotros! Escríbenos*; [Name], how are you feeling? We know the first 24 hours are the most difficult, remember that you can count on us! Text us
Prescheduled standard messages	Maintenance	28 days	3 to 4 messages a day	*Dejar de fumar es una decisión que se toma todos los días. Reafírmala cada mañana. ¡Lo estás logrando!*; Quitting smoking is a decision you make every day. Reaffirm it each morning. You are doing it!; *[Nombre], ¿estás disfrutando esta nueva etapa de tu vida? Cuéntanos lo que más te gusta de haber dejado de fumar*; [Name], are you enjoying this new stage in your life? Tell us what you like the most about no longer smoking.; *¿Ya publicaste en Facebook que dejaste de fumar? ¡Te sorprenderá ver la respuesta positiva de todos!*; Have you posted on Facebook that you quit smoking? You will be surprised to see the positive responses from everyone!; *¿Ya probaste tu platillo favorito ahora que dejaste el cigarro? Redescubre su sabor, ¡disfrútalo!*; Have you tried your favorite dish now that you quit smoking? Rediscover its flavor. Enjoy it!
Prescheduled standard messages	Relapse prevention	56 days	1 to 2 messages a day	*Hoy puedes decir: “¡Soy [Nombre] y llevo 35 días sin fumar! ¡Sigo adelante!*; Today you can say, ”I am [Name] and I have gone 35 days without smoking! I can keep going!“; *¡Felicidades [Nombre]! Ahora que dejaste de fumar, es menos probable que tus hijos(as) lo hagan. ¡Sigue cuidando tu futuro y el de los tuyos!*; Congratulations [Name]! Now that you stopped smoking, it is less likely that your children will do it. Keep taking care of your future and the future of your loved ones!; *[Nombre], planea un bonito fin de semana con las personas que más quieres. ¡6 semanas sin fumar es un gran motivo para celebrar!*; [Name], plan a nice weekend with the people you love the most. 6 weeks without smoking is a great reason to celebrate!
Prescheduled standard messages	Relapse	3 days	4 messages a day	*Recaer no significa que has perdido la batalla, recaer nos da la oportunidad de aprender y salir victoriosos. ¡Inténtalo una vez más!*; To relapse does not mean you have lost the battle; to relapse gives you the opportunity to learn and become victorious. Try it one more time!; *[Nombre], ¿qué te hace fumar? Cuéntanos. Juntos encontraremos nuevas soluciones para dejar el tabaco;* [Name], what makes you smoke? Tell us. We can find new solutions together to quit tobacco
Keyword-triggered standard messages	Family	Per participant request	14 messages	*Dejar de fumar vale la pena por ti y por tus seres queridos. [Nombre], ¡te felicitamos por tu esfuerzo! ¡Sigue adelante!;* Quitting smoking is worth it for the sake of you and your loved ones. [Name], we congratulate for your effort! Keep going!; * [Nombre], dejar de fumar también demuestra amor a tu familia. Sigue así, ¡vas muy bien!;* [Name], quitting smoking also shows love for your family. Keep going; you are doing great!
Keyword-triggered standard messages	Crave	Per participant request	10 messages	*Deja lo que estás haciendo y camina por 5 minutos. Te distraerás y el antojo desaparecerá;* Drop what you are doing and walk for 5 minutes. It will distract you and the cravings will go away.; *[Nombre], piensa en lo lejos que has llegado. No vale la pena volver a fumar, ¡sigue adelante!;* [Name], think of how far you have come. It is not worth it to smoke again, keep going!
Keyword-triggered standard messages	Stress	Per participant request	11 messages	*Inhala profundo por 3 segundos, aguanta el aire por otros 3, y exhala por 6 segundos. Mientras respiras hondo, imagina que estás en un lugar bello y tranquilo. Repítelo, ¡esto te relajará!;* Inhale deeply for 3 seconds, hold your breath for another 3 seconds, and exhale for 6 seconds. While you breathe deeply, imagine that you are in a beautiful and peaceful place. Repeat it; this will relax you!; *[Nombre], escucha tu música favorita, te servirá para controlar el estrés. ¡Será de mucha ayuda!;* [Name], listen to your favorite music, it will help you manage stress. It will be a great help!
Keyword-triggered standard messages	Advice	Per participant request	9 messages	*¡Mantente ocupado! Hay tantas cosas que puedes hacer sin fumar: ir al cine, ir por un café, leer, bailar, ejercitarse;* Keep yourself busy! There are so many thing that you can do without smoking: go to the movies, go for a coffee, read, dance, and exercise; *[Nombre], caminar al menos 15 minutos al día es excelente durante el proceso para dejar de fumar, ¡muévete!;* [Name], walking at least 15 minutes a day is excellent during the quitting process, get moving!
Keyword-triggered standard messages	Sadness	Per participant request	24 messages	*[Nombre], ¿sabías que el ejercicio mejora tu estado de ánimo? El ejercicio te hará sentir bien física y mentalmente. Camina, baila o ve al gimnasio. Te ayudará a conseguir tu objetivo;* [Name], did you know exercising improves your mood? Exercising will make you feel better, both physically and mentally. Walk, dance, or go to the gym. It will help you reach your goals; * [Nombre], la gente que nos rodea influye en nuestro estado de ánimo. Trata de rodearte de personas positivas, que te hagan sentir bien;* [Name], people around us affect our mood. Try to surround yourself with positive people that make you feel good

#### Nicotine Replacement Therapy

The choice of pharmacotherapy followed the practice guidelines for treating smokers in Mexico [16,17]. Only daily smokers who smoked 6 or more CPD were eligible to use nicotine patches. Nicotine patches were contraindicated in participants who (1) had a heart attack in the last 2 months, (2) had a stroke in the last 6 months, (3) have been diagnosed with an arrhythmia or tachycardia, (4) have uncontrolled hypertension, and (5) were using warfarin. Nicotine patches were not offered to these participants. Participants who smoked 10 or more CPD and had no contraindications were offered to use 10 weeks of nicotine patches: 21-mg nicotine patches to be used during the first 6 weeks, followed by 14-mg nicotine patches for 2 weeks, and 7-mg patches for the last 2 weeks. Participants who smoked between 6 and 9 CPD and had no contraindications were offered to use 8 weeks of nicotine patches: 14-mg nicotine patches to be used during the first 6 weeks, followed by 7-mg patches for the last 2 weeks. The NRT was provided in 2 phases. At baseline, each participant received a 4-week supply if they were eligible and interested in using it. Beginning at the second week of the intervention, participants received text message queries to see if they were interested in receiving more NRT. If a participant indicated such an interest, a 4- or 6-week supply was shipped to their home. Participants were prompted to start using their nicotine patches on their selected quit date.

#### Gateway Infrastructure

To implement the text messages system, we worked with *Agile Health Inc* [42], a text messaging health company, which hosted the software to interface with the *Vive sin Tabaco... ¡Decídete!* tablet-based software [34]. We utilized *Agile Health* ’s platform and application program interface to create a customized system to support the smoking cessation program. The platform allowed the counselor to monitor the text messages sent by the participants and categorize the messages using keywords (which triggered an automatic response) and counselor-required messages. The platform allowed the counselor to interact with participants while being able to see the text message history and other participant information such as age, gender, and the date they started the intervention. Text messages were delivered through *Auronix* [43], a text message gateway in Mexico identified through market research and technical testing. *Auronix* ’s service provider had the capacity to (1) engage bidirectional real-time communication through a short code, (2) connect to all carriers in Mexico, (3) receive participants’ text messages containing special characters used in Spanish (ñ á é í ó ú ¡ ¿), and (4) send a large number of text messages from *Agile Health* ’s platform.

### Measures

The in-person baseline survey assessed sociodemographic variables such as age, gender, education level, marital status, and type of health insurance. Other variables collected were physical nicotine dependence (the Fagerström Test for Nicotine Dependence [44]—a 6-item test that evaluates the quantity of cigarette consumption, the compulsion to use, and the dependence), the number of cigarettes smoked per day, and the number of previous quit attempts. We also assessed the frequency of messages that participants sent to the program, including the use of keywords, across each stage of the intervention. At 12 weeks after enrollment, an in-person follow-up survey was conducted by trained research staff and biological samples were collected to verify cessation status. Acceptability measures included satisfaction questions such as “How satisfied are you with the smoking cessation text message program?”

### Outcomes

The primary outcome was cotinine-verified 7-day point prevalence abstinence (no cigarettes in the past 7 days) at 12 weeks. This was biochemically verified using urinary cotinine testing, with a cutoff of 200 ng/ml cotinine [45,46]. If the participant was still using NRT, exhaled carbon monoxide, with a cutoff of 6 ppm [45], was used to verify smoking abstinence. The secondary outcomes were acceptability of the program and text messaging interactivity.

### Data Analysis

We calculated simple frequencies for categorical variables and means and SDs for continuous variables. The primary analysis on cessation was conducted using an intention-to-treat analysis, in which participants lost to follow-up are considered smokers.

## Results

### Participant Recruitment and Characteristics

**Figure 1:**
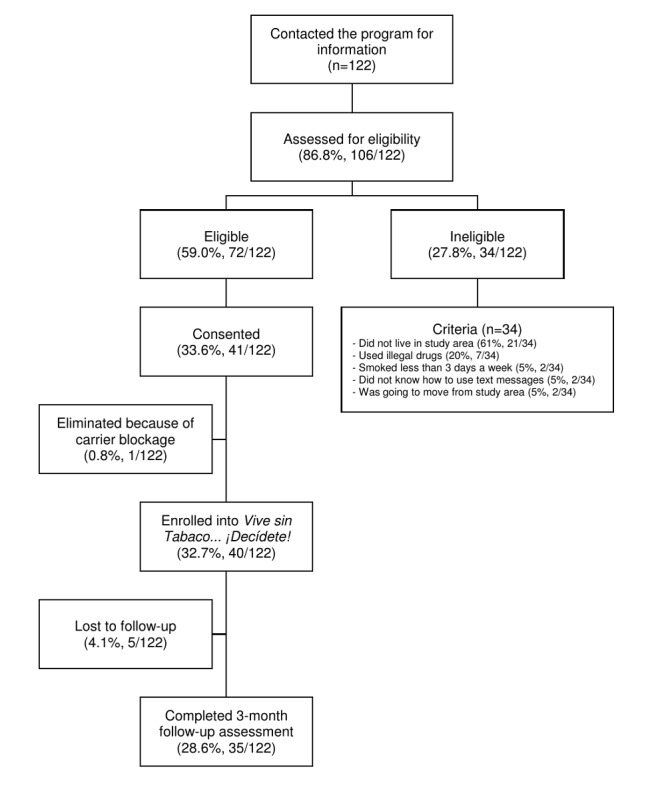
Vive Sin Tabaco… ¡Decídete! intervention flow.

During a single week of recruitment, 122 smokers contacted the study personnel via phone or email for information; among them, 106 were contacted and assessed for eligibility by telephone and 72 were identified as eligible for the study. Overall, 41 smokers consented to participate and completed the baseline assessment in person; 1 smoker was removed from the study because of a carrier blockage that could not be solved, resulting in 40 smokers enrolled in the study (Figure 1).

Participants’ age at baseline ranged from 20 to 59 years (mean 36.0, SD 10.7); 65% (26/40) of the participants were men, 50% (20/40) were single, 62% (25/40) had college or postgraduate education, and 80% (32/40) had health insurance coverage. Most participants smoked daily (95%, 38/40) and were interested in quitting in the next 7 days (95%, 38/40). Half of the participants were light smokers (smoked 10 or less CPD) and, according to the Fagerström test, 70% (28/40) of the participants reported low levels of nicotine dependence (Table 2).

**Table 2:** Baseline characteristics of participants (n=40).

Profile characteristics		Statistics
Age, mean (SD)		36.0 (10.7)
**Sex,** **n (%)**		
	Men	26 (65)
**Education level, n (%)**		
	Less than high school graduate	3 (7)
	High school graduate	7 (17)
	Technical school	5 (12)
	College graduate	18 (45)
	Postgraduate	7 (17)
**Marital status, n (%)**		
	Married or cohabitating	16 (40)
	Single	20 (50)
	Divorced, separated, or widowed	4 (10)
**Health coverage, n (%)**		
	Instituto Mexicano del Seguro Social (English: Mexican Social Security Institute)	25 (62)
	Instituto de Seguridad y Servicios Sociales de los Trabajadores del Estado (English: Institute for Social Security and Services for State Workers)	7 (17)
	None	8 (20)
**Smoking pattern, n (%)**		
	Nondaily	2 (5)
	Daily, 1-9 CPD^a^	20 (50)
	Daily, 10-19 CPD	12 (30)
	Daily, 20 or more CPD	6 (15)
**Fagerström test for nicotine dependence, n (%)**		
	Low dependence	28 (70)
	Moderate dependence	11 (27)
	High dependence	1 (2)
**Quit attempt in the previous year** **, n (%)**		
	Yes	26 (65)
	No	14 (35)

^a^CPD: cigarettes per day.

### Text Messaging Utilization

**Figure 2:**
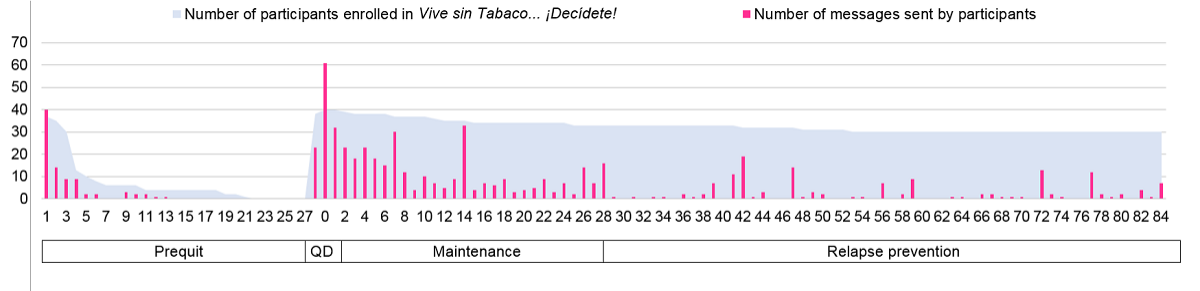
Text messages interaction by participants during the intervention. QD: quit day.

Participants received approximately 180 automated messages during the 12 weeks; none of the participants texted the word STOP to disenroll from the program. During the 12-week intervention period, participants sent 843 text messages, an average of 21 text messages per participant (SD 17.62). Of the 843 messages that participants sent to the program, only 96 (11.3%) used keywords. Participants varied in the frequency of sending text messages: 3 (7%, 3/40) never interacted with the program, 16 (40%, 16/40) had low interaction (1-9 messages), 17 (37%, 17/40) had medium interaction (10-49 messages), and 4 (10%, 4/40) had high interaction (>50 messages). Interaction varied across the different stages of the program (Figure 2). Interaction was very high at the beginning of the intervention and on the quit date, decreasing progressively as the program continued except for spikes on days 7, 14, 28, 42, 56, and 77 after the quit date. These spikes were because of smoking status being assessed on those days via text message. Overall, 15 (37%, 15/40) participants notified the program that they had relapsed and 7 (17%, 7/4-) set up a new quit date.

### Pharmacotherapy Utilization

**Figure 3:**
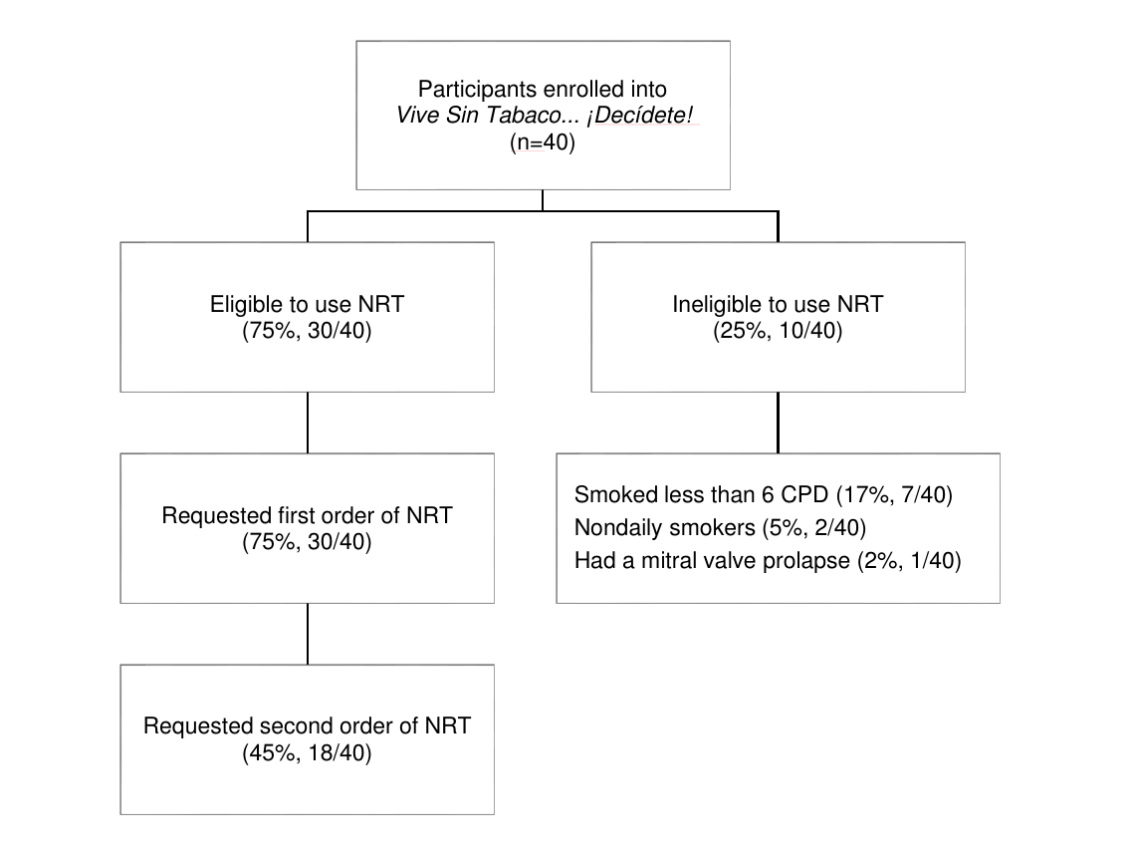
Utilization of nicotine replacement therapy (NRT) during the intervention. CPD: cigarettes per day.

Three-quarters (75%, 30/40) of the participants were eligible to use NRT, all of whom requested an initial supply of NRT. Of these 30 participants who requested NRT at baseline, 18 (60%) requested a refill at 4 weeks (Figure 3).

### Cessation, Retention, and Satisfaction

At 12 weeks, 16 participants (40%, 16/40) were biochemically verified abstinent using intent-to-treat analysis (Table 3). The follow up-rate at 12 weeks was 87.5%. Of the participants who completed the follow-up assessment, most (85.7%) reported being very satisfied or extremely satisfied with the program. In addition, 17 participants (48.5%) reported not being able to send text messages at some point of the intervention because of not having enough credit on their cell phones.

**Table 3:** The 3-month follow-up outcomes.

Outcome		n (%)
**Smoking Abstinence (n=40)^a^**		
	Self-reported 7-days smoking abstinence	18 (45)
	Biochemically verified abstinence urine cotinine (≤200 ng/ml)	15 (37)
	Biochemically verified abstinence exhaled carbon monoxide (≤6 ppm)	1 (2)
**Satisfaction (n=35)**		
	Extremely satisfied	13 (37)
	Very satisfied	17 (48)
	Satisfied	5 (14)

^a^Intention-to-treat analysis was used.

## Discussion

### Principal Findings

To the best of our knowledge, *Vive sin Tabaco... ¡Decídete!* is the first smoking cessation mobile intervention specifically tailored to the needs of Mexican smokers. The program has a unique 2-component platform that includes a tablet-based program at baseline to collect information used to develop a personalized quit plan and the delivery of the 12-week messaging program. This system generated substantial interest among smokers, as indicated by the rapid recruitment of our sample within a single week. The program was well received by the participants, most of whom engaged in high levels of interactivity with the program (eg, bidirectional messaging) and indicated high levels of participant satisfaction. The participants also expressed a high level of interest in using NRT in conjunction with the text messaging program. Although we did not assess medication adherence, the vast majority of participants used the text messaging program to request additional NRT, suggesting that most participants completed at least a 4-week course of therapy. The 40% rate of smoking cessation seen at week 12 (end of treatment) appears promising and is in line with end-of-treatment cessation rates seen in trials of NRT that used substantial in-person counseling.

### Implications for Future Research

A smoking cessation mobile intervention can only be effective and sustainable if it is properly deployed in an environment that reaches a large number of smokers in need of evidence-based services. In Mexico, the most logical setting for the deployment of *Vive sin Tabaco... ¡Decídete!* is within the universal health care system, which is founded on a network of comprehensive primary care clinics [47,48]. Primary health within Mexico’s universal health care system follows established guidelines for the identification and treatment of smokers [16,17]. However, implementation of these guidelines has been limited because of the lack of time during routine care, inadequate training of personnel, and competing patient demands [18]. *Vive sin Tabaco... ¡Decídete!* has the potential to overcome these barriers as it was designed for easy integration into primary health centers without disrupting clinical workflows.

The text messaging program appears to be a promising, low-cost alternative to in-person or telephone counseling to prompt smoking cessation, although additional strategies to eliminate the costs incurred by participants generating text messages to interact with the program may be needed. In this study, participants preferred to send their own, self-composed text messages rather than relying on keywords from the program for a response. This suggests that reliance on keywords may be insufficient for smoking cessation counseling via text messaging in Mexico. Hence, there may be additional costs involved in having trained personnel responding to participants’ text messages, as occurred in this study. Participants’ text messages content should be analyzed using qualitative methods to identify common themes. These methods can guide the creation of a categorized codebook that would be able to retrieve and send responses automatically, thus reducing the need for trained personnel responding to self-composed participants’ text messages.

### Limitations

This study had a number of limitations. This was a pilot study and did not have a control group. Due to the small sample size, the results are not generalizable to all Mexican smokers. Follow-up was limited to a single assessment at week 12, when the program ended. Analyses were limited to quantitative assessments of participant interactions. Furthermore, the sample was more highly educated and smoked more heavily than the general population of smokers in Mexico; future research is warranted to determine whether the effectiveness of this type of intervention is generalizable to those who are from lower socioeconomic status groups. Contrary to the US clinical guidelines [49], NRT is contraindicated in Mexico for those who smoke less than 6 cigarettes a day [17], which is a group that represents about 75% of smokers in Mexico [1]. It is possible that the cessation rate in this study could have been higher if light smokers had access to NRT as determined in the US clinical guidelines [49]. Despite these limitations, the study suggests that *Vive sin Tabaco... ¡Decídete!* is highly acceptable and holds promise for further testing, including a cost-effectiveness analysis.

### Conclusions

The *Vive sin Tabaco... ¡Decídete!* smoking cessation mobile intervention was well accepted by participants, generated high satisfaction and frequent 2-way interactivity, and resulted in noteworthy cessation rates at the end of treatment. The program appears to offer a promising strategy for smoking cessation in Mexico, particularly in the context of primary care clinics that could deploy the *Vive sin Tabaco... ¡Decídete!* tablet to assist participant enrollment. Additional testing in a formal randomized clinical trial is needed before widespread dissemination of *Vive sin Tabaco... ¡Decídete!*

## Abbreviations

CPD: cigarettes per day
NRT: nicotine replacement therapy
